# Multidimensional Quality Evaluation of Imaging Performance for Musculoskeletal Ultrasound Computed Tomography System

**DOI:** 10.3390/bioengineering13091094

**Published:** 2026-09-21

**Authors:** Xiaoxue Bai, Li Zhao, Pan Zhou, Ge Bai, Longxin Xiao, Jianan Chen, Ye Yu, Weicheng Yan, Yang Xu

**Affiliations:** 1 Hubei Medical Devices Quality Supervision and Test Institute, Wuhan 430075, China; 2 Wuhan Intelligent OptoElectronics Co., Ltd., Wuhan 430075, China; 3 Department of Biomedical Engineering, School of Life Science and Technology, and Advanced Biomedical Imaging Facility, Huazhong University of Science and Technology, Wuhan 430074, China

**Keywords:** musculoskeletal ultrasound, computed tomography, ultrasound phantom, performance testing, three-dimensional reconstruction

## Abstract

Core parameters of the Musculoskeletal Ultrasound Computed Tomography System (MUCTS), such as spatial resolution, slice thickness, and three-dimensional reconstruction volume error, directly determine imaging performance and clinical diagnostic accuracy. To objectively and systematically assess the imaging quality and diagnostic reliability of this device, this study took the MUCTS developed by Weishi Medical Imaging Co., Ltd., as the test object. Multidimensional performance tests were carried out using standard ultrasound phantoms and polyurethane-based tissue-mimicking musculoskeletal phantoms. Under unified imaging and reconstruction parameters, key indicators, including effective detection radius, axial resolution, lateral resolution, imaging dead zone, slice thickness, geometric position accuracy, and three-dimensional reconstruction volume error, were quantitatively measured, and compliance verification was performed against product technical specifications. The test results demonstrated that the nominal center frequency was 3 MHz with a measured frequency deviation of 3%, and the effective detection radius reached 90 mm. Within the valid imaging range, the axial resolution was 0.5 mm, lateral resolution was 1 mm, imaging dead zone was 1 mm, and slice thickness was 3.2 mm. The relative error of geometric position accuracy was 3%, the relative deviations in perimeter and area measurements were both −1%, and the relative deviation in three-dimensional reconstructed volume was −8%. All indicators satisfied product technical requirements. Qualitative comparative experiments on tissue-mimicking phantoms confirmed that the system could reproduce multiscale and multi-contrast musculoskeletal anatomical features, and the acquired images showed favorable consistency with designed phantom targets. In conclusion, this musculoskeletal ultrasound tomographic system exhibits stable imaging performance and credible quantitative measurements, and can meet clinical and research demands for tomographic imaging and three-dimensional reconstruction of human musculoskeletal tissues.

## 1. Introduction

Musculoskeletal disorders cover acute sports injuries [1,2], tissue degenerative changes [3], chronic cumulative strain [4,5], and postoperative motor dysfunction [6,7]. These clinical conditions have high prevalence with occult early-stage lesions and often accompany dynamic functional impairment, imposing higher requirements on the refinement, dynamism, and quantitation of radiological examinations [8,9]. Conventional imaging modalities have respective limitations. X-ray and CT provide prominent visualization of bony structures, yet suffer from poor soft-tissue discrimination. They fail to identify subclinical injuries such as micro-tears of muscle fibers and minor ligament damage. Additionally, ionizing radiation restricts their application in long-term follow-up [10,11,12,13]. MRI delivers excellent soft-tissue contrast and serves as an essential tool for detailed musculoskeletal evaluation. Nevertheless, the high equipment cost, prolonged scanning time, and fixed patient positioning prevent dynamic imaging under motion conditions, limiting its utility for bedside screening and real-time assessment of sports-related injuries [14,15]. In recent years, intelligent functional materials and additive manufacturing technologies have provided novel technical approaches for the innovative development of the ultrasonic imaging field. For instance, studies have demonstrated the development of deployable medical functional structures suitable for imaging-guided applications by fabricating near-infrared-responsive 4D-printed shape-memory polymer composites, offering cutting-edge insights for the intelligent expansion of new ultrasonic imaging modalities and their application in precision diagnosis and therapy [16].

As a new-generation ultrasound imaging technology, the Musculoskeletal Ultrasound Computed Tomography System (MUCTS) overcomes the drawbacks of limited field-of-view and single-plane scanning in conventional two-dimensional ultrasound [17]. Through full-field acoustic signal acquisition, high-precision spatial registration, and tomographic reconstruction algorithms, MUCTS enables continuous tomographic imaging and three-dimensional visualization of musculoskeletal tissues. It is capable of depicting millimeter-scale fine anatomy and early-stage injuries, compensating for the technical deficiencies of X-ray, CT, and MRI in fine soft-tissue imaging and dynamic detection [8,18]. Currently, MUCTS has been gradually applied in screening, diagnosis, and postoperative rehabilitation assessment for musculoskeletal diseases [19,20,21].

Imaging performance forms the fundamental guarantee for reliable MUCTS outputs and supports quantitative clinical diagnosis. Key parameters jointly govern clinical utility from the perspectives of imaging coverage, spatial resolution, geometric positional accuracy, and three-dimensional reconstruction precision [22,23,24]. The acoustic operating frequency balances tissue penetration depth and image detail resolution [25]; detection radius defines valid imaging coverage for global scanning of musculoskeletal structures; axial and lateral resolutions reflect the capability to resolve tiny anatomical features and early micro-lesions; the near-field dead zone sets the minimum detection limit for superficial tissues; slice thickness determines inter-slice fidelity and suppression of partial-volume artifacts; geometric position accuracy and dimensional measurement deviations in perimeter, area, and reconstructed volume are critical metrics for anatomical localization and quantitative evaluation, constituting the basis for refined clinical analysis [26,27,28]. Therefore, multi-parameter and multi-scenario systematic performance evaluation is of great research and clinical significance for establishing quality-control criteria and safeguarding diagnostic accuracy.

Different from conventional evaluation schemes for clinical B-mode ultrasound, the proposed MUCTS performance framework is specifically tailored to its integrated transceiving and three-dimensional tomographic imaging principle. Conventional commercial ultrasound phantoms are only applicable to single-sided reflection scanning and limited to two-dimensional image assessment, making them incapable of characterizing tomographic imaging performance. By contrast, the customized phantoms developed by the Institute of Acoustics, Chinese Academy of Sciences, support multi-angle transmission–reflection acquisition and contain built-in three-dimensional reference targets. This design enables comprehensive quantification of both routine two-dimensional imaging metrics and unique tomographic parameters, including volumetric reconstruction accuracy and depth-dependent imaging degradation, thereby overcoming the inherent limitations of conventional B-mode ultrasound evaluation protocols.

## 2. Materials and Methods

### 2.1. Principles and Imaging Quality Requirements of MUCTS

#### 2.1.1. Imaging Principles of MUCTS

**Figure 1:**
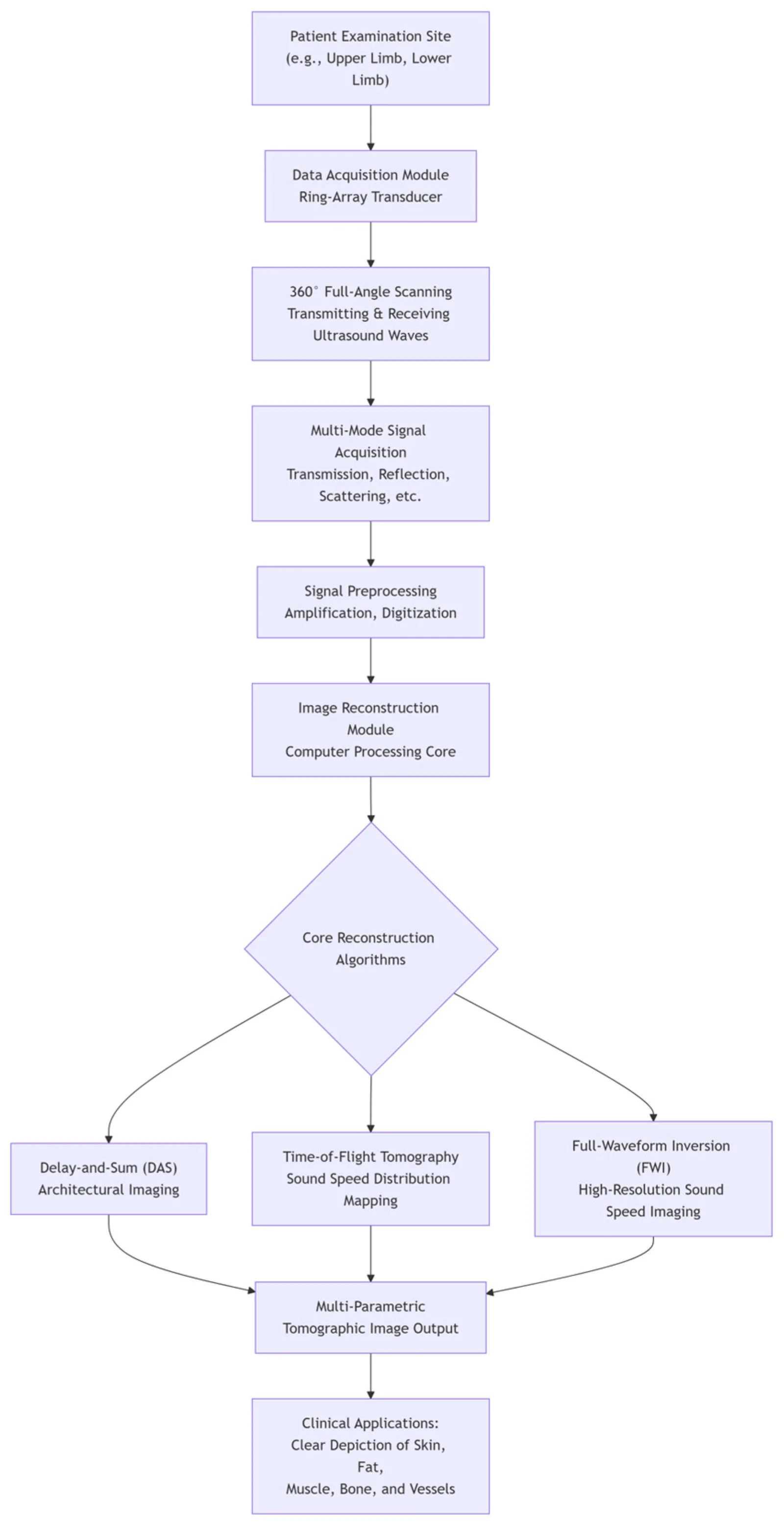
Workflow diagram of MUCTS.

MUCTS operates based on reflection, scattering, and attenuation effects of ultrasonic waves propagating within biological tissues. High-frequency ultrasonic transducers emit pulsed ultrasound towards tissues. Acoustic waves are reflected and scattered at interfaces with discontinuous acoustic impedance, including muscles, tendons, ligaments, fasciae, and bone cortices. The ring-array transducer acquires full-field echo signals, which go through pre-processing procedures such as signal amplification, noise reduction and filtering, time-gain compensation, and phase correction. Combined with high-precision spatial registration, tomographic image reconstruction and three-dimensional model generation are implemented to visualize musculoskeletal anatomy and pathological information (Figure 1). Compared with conventional two-dimensional ultrasound, MUCTS offers full-field view, continuous inter-slice information, and superior tissue restoration. It can faithfully reproduce spatial adjacency among musculoskeletal components and is well-suited for the comprehensive assessment of complex musculoskeletal anatomy [29].

#### 2.1.2. Technical Challenges and Performance Requirements for MUCTS

Human musculoskeletal systems possess a sophisticated layered architecture with subtle acoustic impedance differences among soft tissues and wide anatomical depth variation, posing greater imaging challenges than conventional ultrasound examinations of the abdomen or cardiovascular system. Four major performance requirements are raised for MUCTS: First, high spatial resolution is required to identify micro-structures such as muscle fibers, tendon sheaths, and peripheral nerve endings ranging from 0.1 to 1 mm for detection of early micro-injuries. Second, favorable contrast resolution is needed to distinguish subtle acoustic differences among soft tissues and detect occult lesions, including tissue oedema and micro-tears. Third, global imaging uniformity is necessary to balance high-resolution visualization of superficial layers and compensation for signal attenuation in deep regions, mitigating brightness and contrast degradation across depth. Fourth, artifact suppression is essential to reduce interference from side-lobe artifacts, multiple-reflection artifacts, and tomographic misregistration on image interpretation [30,31]. Musculoskeletal ultrasound image quality depends on the synergistic performance of multiple parameters; deficiency in any single metric may degrade overall image quality and hence, multi-parameter combined quality control is indispensable.

### 2.2. Core Hardware Parameters and Imaging Advantages of the Transducer

Ultrasonic transducers serve as the acoustic front-end of ultrasound systems. Beam-focusing performance, transmission precision, and echo sensitivity directly determine overall imaging quality [31]. The tested MUCTS developed by Weishi Medical Imaging Co., Ltd. (Wuhan, China), has a nominal center frequency of 3 MHz, effective bandwidth of 1.5–4.5 MHz, total element count of 2048, and 1024 independent signal channels. Such hardware configuration balances penetration depth and enables the coverage of deep muscle groups, peri-articular soft tissues around hip and knee joints, as well as complex periosseous anatomy. High-density array elements optimize beam focusing, restrain beam divergence, and reduce scanning dead zones and signal distortion. Multi-channel parallel acquisition improves spatial sampling density and signal-acquisition efficiency, guarantees uniformity and continuity of tomographic scanning, mitigates inter-slice artifacts, and provides solid hardware support for full-field high-precision musculoskeletal imaging.

### 2.3. Multidimensional Quality Evaluation of MUCTS Imaging Performance

#### Experimental Equipment and Environmental Conditions

The tested MUCTS consists of a main control console, automatic scanning unit, dedicated examination couch, and supporting image-analysis software, equipped with a ring-array ultrasonic transducer. Its nominal operating frequency is 3.0 MHz, rated voltage is 220 V ± 10%, and rated input power is 1500 VA. All tests were performed in accordance with equipment specifications and product technical requirements. Environmental conditions were controlled as: temperature, 10–30 °C; relative humidity, 15–70% R.H. (non-condensing); atmospheric pressure, 86–106 kPa. All phantom imaging experiments were performed under the custom-scan mode of the MUCTS. The scan step was set to 1 and the scan travel was set to 1, generating one automatic scan layer. The imaging range was 220, and the sound speed was adjusted within 1450–1550. The imaging quality level was set to 7 with a TGC coefficient of 0.0003. The scan starting coordinate was registered to the real-time probe position. Scanning speed and global system gain followed factory defaults and were not manually modified in this study. Image reconstruction and filtering were performed using the manufacturer-provided default built-in pipelines under the above acquisition settings, with no additional custom filtering or reconstruction tuning applied. The device completed self-check and sufficient warm-up before formal testing after stable operation was confirmed.

### 2.4. Experimental Protocol for Standard Ultrasound Phantoms and Tissue-Mimicking Phantoms

A series of standard ultrasound phantoms manufactured by the Institute of Acoustics, Chinese Academy of Sciences, were adopted to establish the multidimensional evaluation system, including a KS107-BHF resolution phantom, a KS107-BHJ uniformity phantom, a KS107-BHQ slice-thickness phantom, and a KS107-BH3D three-dimensional volumetric reconstruction phantom. The KS107-BHF resolution body model enables quantitative assessment of axial and lateral spatial resolution, corresponding to the capability for identifying micro-structures such as fine tendons, thin fascial layers, and nerve endings in clinical musculoskeletal imaging; the KS107-BHJ uniformity body model evaluates the uniformity of the acoustic field distribution, image signal-to-noise ratio, and artifact-suppression level, reflecting the imaging stability of the device when imaging homogeneous soft tissues across deep and superficial muscle groups; the KS107-BHQ slice-thickness body model precisely measures the thickness of cross-sectional slices and quantifies interlayer signal crosstalk and partial volume effects, aligning with the cross-sectional imaging characteristics of multi-layered, interlaced musculoskeletal soft tissues; the KS107-BH3D three-dimensional volumetric reconstruction body model is specifically designed to validate the accuracy of three-dimensional geometric reconstruction and volume quantification, supporting clinical measurement of the lesion volume, assessment of injury extent, and quantitative postoperative follow-up.

Thermostatted, fully degassed deionized water was filled into the coupling container prior to experiments. Each phantom was positioned at the imaging center with precise alignment. Scanning and reconstruction parameters were unified for tomographic acquisition and post-processing. Measured performance metrics were compared against product technical specifications for compliance analysis. The performance evaluation referred to the manufacturer-provided product specification of the tested device. Reference standards, acceptance limits, and evaluation basis for key parameters are summarized in Table 1.

**Figure 2:**
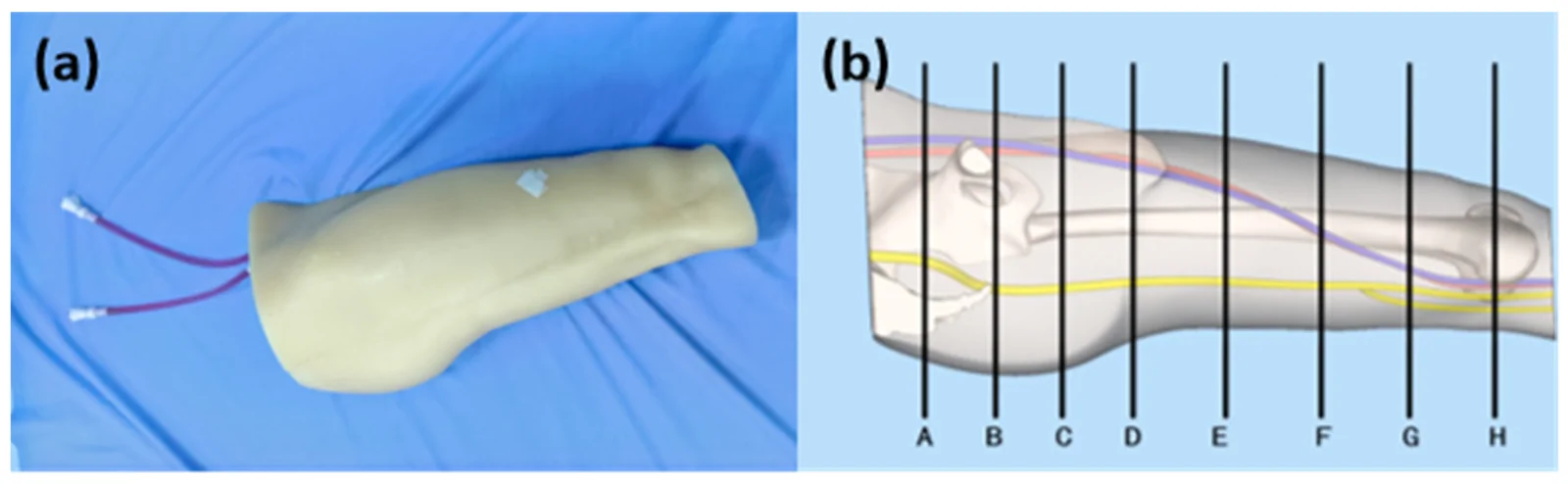
Tissue-mimicking phantom simulating adult thigh: (**a**) physical phantom; (**b**) anatomical schematic diagram—planes A–H mark consecutive transverse tomographic scanning positions. Red line target represent arterie, blue line targets represent vein, and yellow line targets represent nerve.

To simulate acoustic environments of human musculoskeletal tissues and evaluate clinical adaptability, qualitative imaging tests were further conducted using a polyurethane-based composite tissue-mimicking phantom developed by Guizhou Mosheng Medical Technology (Figure 2). This phantom mimics adult thigh anatomy with a sound velocity of 1540 ± 10 m/s and an acoustic attenuation coefficient of 0.5–0.7 dB/cm/MHz. Its soft-tissue scattering characteristics approximate human tissues. Embedded targets simulating bones, nerves, and blood vessels reproduce high-/low-acoustic-impedance interfaces and delicate soft-tissue structures of musculoskeletal systems. This phantom reproduces clinical structures such as large and deep muscle groups and peri-femoral bone-related tissues, realistically reflecting imaging conditions in obese patients, deep chronic strain, and concealed large-joint injuries. Planes A–H in Figure 2b represent eight consecutive transverse tomographic slices. Scanning range, slice number, and reconstruction precision were fixed throughout experiments. The phantom was secured inside the coupling container with spatial registration to ensure all targets fell within the valid field-of-view. Multi-slice tomographic images were acquired, and measured outputs were compared with phantom-designed parameters to assess imaging fidelity, capability for resolving micro-structures, and measurement reliability under human-tissue-mimicking acoustic conditions.

Thus, standard test specimens establish the upper limit and traceability of device performance under an ideal uniform environment, while the thigh prosthetic validates its clinical effectiveness under conditions that approach the complex acoustic environment of the human body; this two-tiered, complementary approach ensures that the assessment results are both accurate and applicable to clinical musculoskeletal practice.

## 3. Results

### 3.1. Quantitative Test Results Obtained from the Standard Ultrasound Phantoms

**Figure 3:**
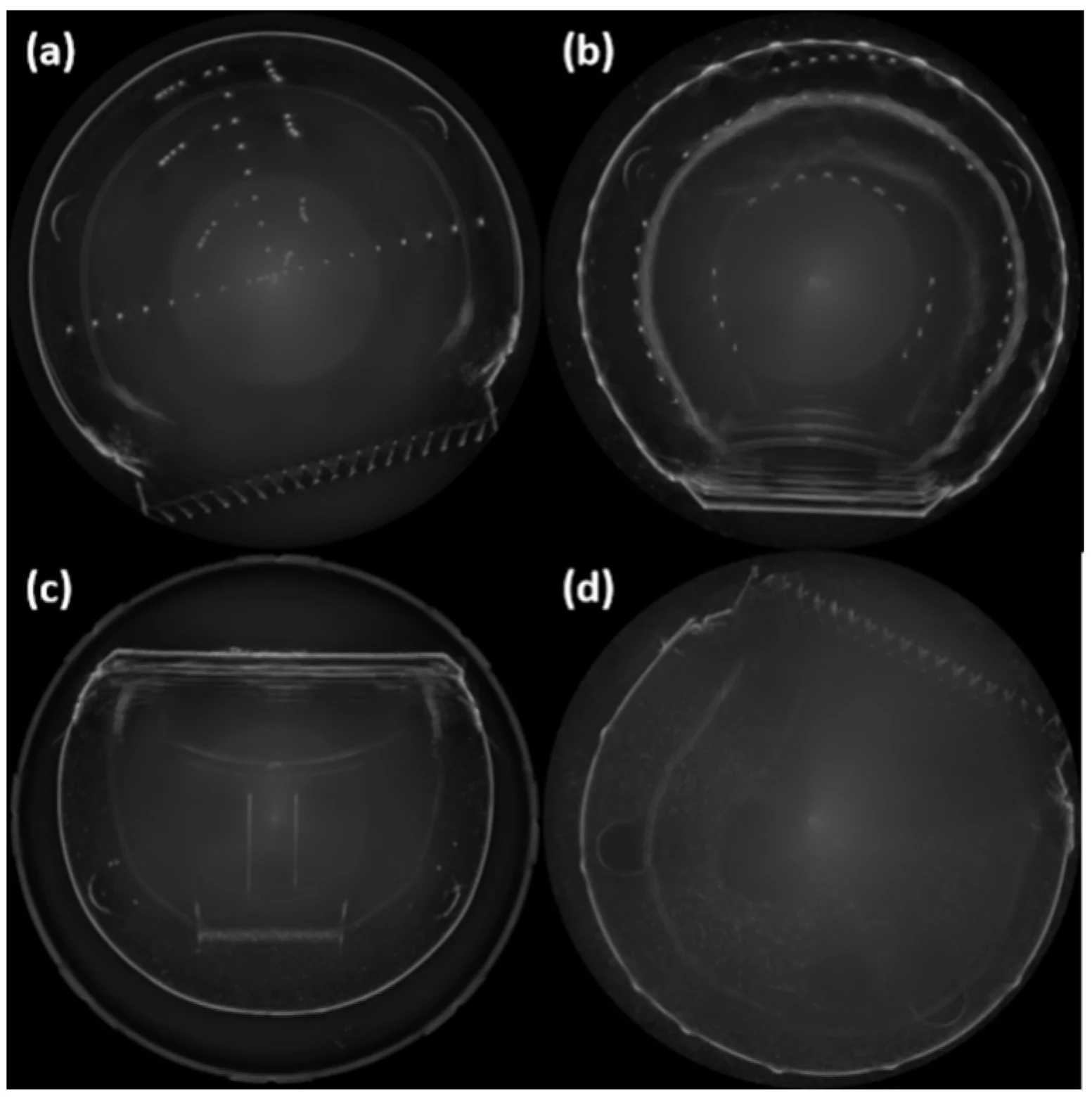
MUCTS tomographic images of standard phantoms: (**a**) KS107-BHF resolution phantom; (**b**) KS107-BHJ uniformity phantom; (**c**) KS107-BHQ slice-thickness phantom; (**d**) KS107-BH3D three-dimensional volumetric reconstruction phantom.

Representative images of standard phantoms are presented in Figure 3. Each performance indicator measured on the standard ultrasound phantom was obtained from eight independent replicate tests, as outlined in Table 2. All image-acquisition and parameter-measurement procedures were completed by a single experimenter to minimize extra variability introduced by manual operation, thus guaranteeing repeatability and internal reproducibility of the experimental results. The measured slice thickness was 3.03 mm (SD: 0.12 mm; 95% CI: 2.93–3.13 mm). Regarding geometric position accuracy, the mean longitudinal and transverse position deviations were 1.50% (SD: 1.31%; 95% CI: 0.40–2.59%) and −0.38% (SD: 2.45%; 95% CI: −2.42–1.67%), respectively. The mean reflection (echo) image perimeter and area deviations were −0.07% (SD: 0.73%; 95% CI: −0.68–0.54%) and 0.48% (SD: 0.50%; 95% CI: 0.07–0.90%), respectively. For the three-dimensional (3D) reconstruction volume deviation, the mean deviations in the large-follicle and small-follicle subgroups were −0.99% (SD: 6.25%; 95% CI: −6.22–4.23%) and −0.16% (SD: 6.91%; 95% CI: −5.93–5.62%), respectively. Since the eight repeated measurements of detection radius and imaging resolution yielded consistent results, the individual raw data are not presented here; the detailed results are shown in Table 3. All metrics exhibited small systematic biases and narrow 95% confidence intervals, indicating that the proposed method offers high measurement accuracy and good reproducibility.

To evaluate the imaging and measurement performance of the proposed method, the inspection data of the conventional B-mode ultrasound system and the MUCTS were compared (Table 3). The results demonstrate that the MUCTS exhibits evident advantages over the conventional B-mode ultrasound across several key indicators. Regarding slice thickness of the reflection image, the conventional B-mode ultrasound measured 6 mm, exceeding the standard upper limit of ≤3.5 mm (failing to meet the requirement), whereas the MUCTS measured 3.2 mm, precisely satisfying the standard; this represents the most notable difference and directly reflects the improved tomographic resolution of the MUCTS. In terms of geometric position accuracy, the MUCTS achieved 3%, markedly better than the 5% of the conventional B-mode ultrasound and well below the 15% standard limit. For the 3D reconstruction volume calculation deviation, the MUCTS exhibited −8%, considerably closer to zero than the −18% of the conventional B-mode ultrasound, indicating higher accuracy in volumetric reconstruction. In addition, both systems met the standards for detection radius (90 mm), axial and lateral resolutions (0.5–1 mm), and blind zone (1 mm), while the MUCTS also demonstrated a more symmetric perimeter and area measurement deviation (−1%). Overall, the MUCTS met the standard requirements in all inspection items and showed significant advantages over the conventional B-mode ultrasound in core metrics—including slice thickness, geometric position accuracy, and 3D reconstruction volume deviation—reflecting higher imaging resolution and measurement accuracy.

### 3.2. Qualitative Imaging Results of the Tissue-Mimicking Phantom

**Figure 4:**
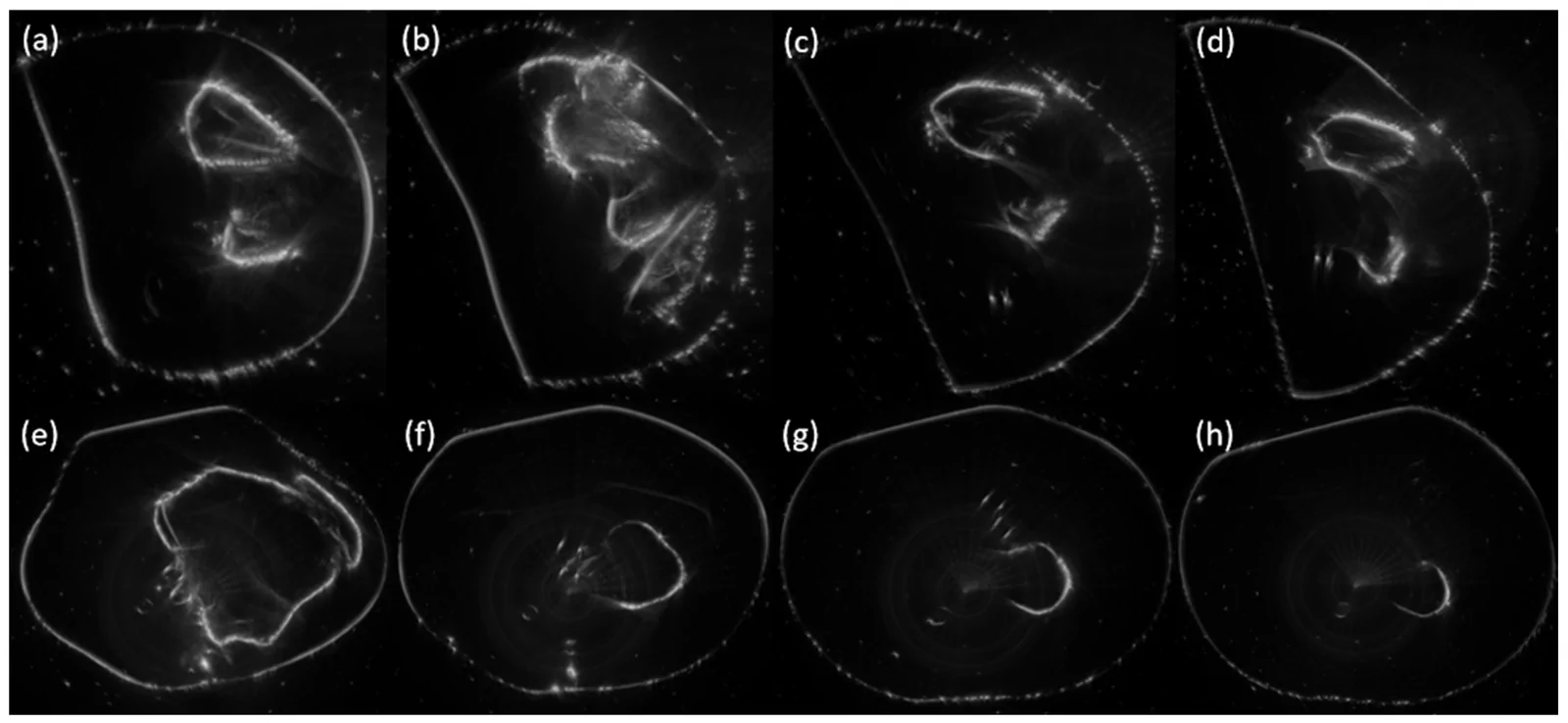
Transverse ultrasound images of tissue-mimicking thigh phantom acquired by MUCTS: (**a**–**h**) correspond sequentially to slices A–H shown in Figure 2b.

Transverse images of the tissue-mimicking thigh phantom are shown in Figure 4, where Figure 4a–h sequentially correspond to slices A–H in Figure 2b, enabling sequential observation from the proximal hip towards the distal femur (knee joint). Spatial positions of bony structures, the simulated sciatic nerve, and the vascular bundle showed good consistency between anatomical schematic diagrams and ultrasound tomograms. At proximal levels (A–D, Figure 4a–d), contours of the acetabulum and proximal femur were clearly visualized. The sciatic nerve–vascular complex coursed within the posterolateral soft-tissue space of the femur, consistent with spatial distribution in anatomical models. During distal scanning (E–H, Figure 4e–h), acetabular anatomy gradually faded and images transitioned to cross-sections of femoral diaphysis. The main trunk of the simulated sciatic nerve ran posterior to the femur with reduced bundle diameter and emerging branches towards distal ends. Morphological evolution agreed well with schematic diagrams.

Discrepancies stem from physical imaging mechanisms. Schematic diagrams are idealized renderings with sharp boundaries for nerves, vessels, and bones. In ultrasound images, bone cortices manifest as hyperechoic bright contours, whereas nerves and blood vessels are identified by echo features combined with anatomical landmarks. Owing to ultrasound scattering noise and acoustic-shadow artifacts, soft-tissue boundaries appear less distinct than those in ideal schematics. The system can reproduce tomographic courses of simulated sciatic nerve and adjacent vessels along the femur and demonstrates the capability for anatomical visualization.

Qualitative observations also revealed imaging limitations. Side-lobe tails and multiple-reflection artifacts appeared at high-impedance interfaces such as bone cortices. Target contours of small-sized simulated nerves and vessels exhibited serration, discontinuity, and marginal blurring. Image contrast degraded with increased penetration depth, and discrete scattering noise existed in homogeneous background regions, interfering with visual identification of tiny low-contrast targets. Overall, imaging performance was superior for large-sized structures with high acoustic-impedance differences, whereas recognition of delicate low-contrast soft-tissue structures remains to be improved.

## 4. Discussion

In this study, standard ultrasound phantoms and tissue-mimicking musculoskeletal phantoms were employed to evaluate MUCTS performance across multiple dimensions, including acoustic parameters, two-dimensional image quality, geometric localization, two-dimensional measurement, and three-dimensional reconstruction. Quantitative results verified that resolution, detection coverage, and geometric and volumetric measurement accuracy of the tested device meet product specifications. The 90 mm effective detection radius enables full-range coverage of superficial and deep musculoskeletal tissues and satisfies imaging and quantitative assessment requirements for most macroscopic musculoskeletal structures. The 3D volumetric reconstruction yielded a steady negative bias of −8%. This systematic underestimation is primarily attributed to the partial volume effect caused by the fixed 3.2 mm slice thickness, blurry soft-tissue boundaries, and contour smoothing during 3D interpolation. Statistical analysis confirmed low data dispersion, indicating high measurement repeatability and stable systematic deviation rather than random error.

Experiments on tissue-mimicking phantoms uncovered existing limitations. Despite high imaging fidelity for high-contrast targets such as bones and large arteries, tiny low-contrast structures including small nerves and delicate fasciae suffered from discontinuous and blurry contours that were jointly induced by ultrasound attenuation and strong-interface artifacts, increasing challenges for detecting micro-lesions. These findings suggest that MUCTS is more suitable for the assessment of macroscopic musculoskeletal anatomy. For refined diagnostic scenarios, such as tendon micro-tears and peripheral nerve injuries, further optimization of signal-acquisition strategies, iterative reconstruction algorithms, and artifact-suppression techniques are required to improve imaging quality for deep micro-structures [32,33,34].

The proposed evaluation system integrates objective quantitative metrics and qualitative image interpretation under human-tissue-mimicking conditions. It can be applied for factory acceptance, clinical access inspection, and periodic quality control. It should be noted that the assessment of side-lobe artifacts, scattering noise, and the depiction of low-contrast structures in the present study is restricted to qualitative phantom observations. Although quantitative indices, including SNR, CNR, and imaging uniformity, are well-established for conventional B-mode ultrasound, standardized calculation protocols and ROI selection criteria have not been fully defined for volumetric images reconstructed from MUCTS multi-angle transmission–reflection data. Development of validated, modality-tailored quantitative workflows for these image-quality parameters will be required in future studies to achieve more objective performance characterization for MUCTS. One notable limitation of this study is that all experiments were performed solely on phantoms, without human clinical samples involved. It should be emphasized that the present work represents a controlled pre-clinical performance characterization study. Prior to its formal adoption for routine clinical practice, human-subject-based clinical validation is mandatory, and preliminary clinical investigations toward this goal have been reported in the literature [35]. Future work shall validate the applicability of this evaluation system using large-scale clinical datasets and continuously refine quality-control standards for MUCTS. According to test results, spatial resolution, deep-region image contrast, and artifact-suppression capacity are recommended as key indicators for long-term quality control and algorithmic optimization of such devices.

## 5. Conclusions

This study constructed a multi-dimensional evaluation system covering acoustic performance, two-dimensional imaging quality, geometric localization, and two-dimensional and three-dimensional measurement accuracy, systematically validating the imaging performance of MUCTS. Test results indicated that the 1.5–4.5 MHz broadband scheme balances penetration depth and detail discrimination. The 90 mm effective detection radius achieves full-range coverage of superficial and deep musculoskeletal tissues. All core metrics, including resolution, dead zone, slice thickness, and measurement deviations, satisfy technical specifications. The system delivers stable imaging and credible quantitative measurements, supporting screening, diagnosis, follow-up, and relevant research for musculoskeletal diseases.

All image quality measurements in this study were performed at fixed depths without layered quantitative analysis across depth gradients. In practice, increased penetration depth leads to gradual degradation of resolution and contrast due to the superposition of ultrasonic attenuation, scattering noise, and side-lobe artifacts. Future multi-depth gradient testing is required to establish depth-dependent variation curves of resolution, SNR, and CNR for further quantitative characterization of system performance limitations.

Tests on tissue-mimicking phantoms clarified performance boundaries of the device. Although imaging of large-scale high-contrast anatomical structures performs well, imaging quality for deep tiny low-contrast soft tissues is compromised by attenuation and artifacts, pointing out directions for subsequent algorithmic optimization. The multi-parameter comprehensive evaluation scheme established herein can objectively reflect practical imaging performance of musculoskeletal ultrasound tomographic devices, providing a reference for the construction of quality-control systems and standardized clinical application of this technology.

## Figures and Tables

**Table 1:** Key performance indicators for MUCTS, corresponding standard specifications, acceptance limits, and bases for judgment explanations.

Performance Indicators	Reference Standard Document	Acceptable Limit	Basis for Judgment Explanation
Detection radius	Product technical requirements	≥90 mm	Phantom KS107-BHF, identifiable deepest target wire image
Axial resolution	Product technical requirements	≤1 mm	Phantom KS107-BHF, resolvable minimum axial target wire spacing
Lateral resolution	Product technical requirements	≤1 mm	Phantom KS107BHF, resolvable minimum lateral target wire spacing
Image blind area	Product technical requirements	≤5 mm	Phantom KS107-BHJ, identifiable target depth closest to the phantom scan surface
Slice thickness	Product technical requirements	≤3.5 mm	Phantom KS107-BHQ, thickness of tissue-mimicking material displaying generated information perpendicular to the scan plane
Geometric position accuracy error	Product technical requirements	≤15%	Phantom KS107-BHF, measure set target spacing, and calculate relative error compared to nominal value
Relative deviation in two-dimensional perimeter measurement	Product technical requirements	≤±20%	Phantom KS107-BHJ, measure two-dimensional geometric target perimeter, and calculate deviation compared to nominal value
Relative deviation in two-dimensional area measurement	Product technical requirements	≤±20%	Phantom KS107-BHJ, measure two-dimensional geometric target area, and calculate deviation compared to nominal value
Relative deviation in three-dimensional volumetric reconstruction	Product technical requirements	≤±30%	Phantom KS107-BH3D, measure oval target reconstructed volume, and calculate deviation compared to nominal value

**Table 2:** Descriptive statistics and 95% confidence intervals of quantitative performance indicators measured with the proposed method (*n* = 8 per metric).

Performance Indicators	Sample Size	Mean	Standard Deviation	95%CI Lower Limit	95%CI Upper Limit
Slice thickness (mm)	8	3.03	0.12	2.93	3.13
Longitudinal geometric position accuracy	8	1.50%	1.31%	0.40%	2.59%
Transverse (lateral) geometric position accuracy	8	−0.38%	2.45%	−2.42%	1.67%
Reflection (echo) image perimeter deviation	8	−0.07%	0.73%	−0.68%	0.54%
Reflection (echo) image area deviation	8	0.48%	0.50%	0.07%	0.90%
Reflection image 3D reconstruction volume deviation (large follicles)	8	−0.99%	6.25%	−6.22%	4.23%
Reflection image 3D reconstruction volume deviation (small follicles)	8	−0.16%	6.91%	−5.93%	5.62%

**Table 3:** Comparison of inspection data between the conventional B-mode ultrasound system and the MUCTS.

Inspection Item	Standard Requirement	Conventional B-Mode Ultrasound	MUCTS
Detection radius (mm)	≥90	90	90
Axial resolution of the reflection image (mm)	≤1 (R ≤ 30)	0.5 (R ≤ 30)	0.5 (R ≤ 30)
	≤1 (30 < R ≤ 60)	0.5 (30 < R ≤ 60)	0.5 (30 < R ≤ 60)
	≤1 (60 < R ≤ 90)	0.5 (60 < R ≤ 90)	0.5 (60 < R ≤ 90)
Lateral resolution of the reflection image (mm)	≤1 (R < 30)	0.5 (R < 30)	0.5 (R < 30)
	≤1 (30 ≤ R < 60)	0.5 (30 ≤ R < 60)	0.5 (30 ≤ R < 60)
	≤1 (60 ≤ R ≤ 90)	1 (60 ≤ R ≤ 90)	1 (60 ≤ R ≤ 90)
Blind zone of the reflection image (mm)	≤5	1	1
Slice thickness of the reflection image (mm)	≤3.5	6	3.2
Geometric position accuracy of the reflection image	Should be within 15%	5%	3%
Perimeter and area measurement deviation in the reflection image	Should be within ±20%	Perimeter deviation: 1%	Perimeter deviation: −1%
		Area deviation: 1%	Area deviation: −1%
3D reconstruction volume calculation deviation in the reflection image	Should be within ±30%	−18%	−8%

## Data Availability

The data presented in this study are available upon reasonable request from the corresponding author. The data are not publicly available due to privacy and ethical reasons.

## Abbreviations

The following abbreviations are used in this manuscript:

MUCTS	Musculoskeletal Ultrasound Computed Tomography System
